# The functional relationships between BBS and MKS and their role in regulating trafficking of proteins in the primary cilium

**DOI:** 10.1186/2046-2530-1-S1-P62

**Published:** 2012-11-16

**Authors:** F Bangs, S Goetz, KV Anderson

**Affiliations:** 1 Sloan Kettering Institute, New York, USA

## 

Bardet-Biedl syndrome (BBS) and Meckel-Gruber syndrome (MKS) are genetic disorders caused by disruption of primary cilia. The BBS and MKS proteins are found in two distinct protein complexes but the functional relationships between these two complexes are not clear. Here we analyze double mutant mouse embryos to define these relationships. *Bbs4;Mks1* double mutant embryos show a stronger phenotype than either single mutant, with completely penetrant micropthalmia and polydactyly, loss of ventral neural cell types, and a reduced number of short bulbous primary cilia. This additive phenotype suggests that BBS and MKS function in parallel pathways. We detected accumulation of Smo and abnormal Gli2 localization in *Bbs4;Mks1* double mutant cilia, suggesting that the double mutants have a defect in retrograde trafficking. To define a role of BBS4 or MKS1 in ciliary trafficking, we have generated *Bbs4* or *Mks1* mice that carry mutations in the IFT machinery. Absence of BBS4 did not affect the phenotype of a weak IFT-B mutant or of embryos that lack the retrograde dynein motor, whereas the Mks1 phenotype was stronger when either IFT-B or dynein were mutant. This implies that BBS works together with IFT to regulate ciliary trafficking whereas MKS1 has a parallel role in ciliary function. Together these data suggest that BBS and MKS both have roles in regulating movement of Hh components in the cilium however the way in which they do this is distinct from each other.

